# Older adults’ perspectives on their residential care homes: a case study

**DOI:** 10.3389/fpubh.2026.1698646

**Published:** 2026-02-18

**Authors:** Marta Gil-Lacruz, Eduardo Alberto Leché-Martín, Marcus Alexander Henning, Ana Isabel Gil-Lacruz

**Affiliations:** 1 Faculty of Health Sciences, University of Zaragoza, Zaragoza, Spain; 2 Ayuntamiento de Zaragoza, Zaragoza, Spain; 3 Faculty of Medical and Health Sciences, The University of Auckland, Auckland, New Zealand; 4 Department of Management and Business Organization, Universidad de Zaragoza, Zaragoza, Spain

**Keywords:** care home evaluation quality, care home residents, living environment, quality of life—older adults care home residents, service provision satisfaction

## Abstract

**Introduction:**

Increasing life expectancy highlights the critical need for high-quality older adults care services. This study examined the satisfaction of residents aged 65 and older in a rural care home in Aragón, Spain, focusing on living spaces, personal relationships, and available services. The insights gathered aim to inform future improvements in service provision and policy.

**Methods:**

A case study approach was employed at a private care home in Sabiñánigo, Spain. Forty residents with functional autonomy were included; individuals with severe cognitive or mobility impairments were excluded. Data were collected via direct interviews using a validated, culturally adapted standardized questionnaire. The instrument assessed satisfaction with services (medical, dining, laundry), facilities (shared areas, rooms), participation, and interpersonal relationships. Descriptive statistics, Pearson correlations, and linear regression analysis were used to identify key determinants of overall satisfaction.

**Results:**

The sample predominantly consisted of women over 80 years old, mostly widowed or single, with preserved self-care capabilities. Most residents shared bedrooms and reported positive perceptions of this arrangement. While dining and healthcare services received high satisfaction ratings, storage space and cafeteria vending machine restrictions were negatively perceived. Furthermore, a significant proportion of residents reported a lack of participation in management suggestions. Inferential analysis revealed that food quality, functional status, and educational level significantly influenced overall satisfaction. Specifically, more educated and autonomous residents tended to rate the care home less favorably.

**Discussion:**

Residents generally reported high satisfaction with personal care and their residential lifestyle, particularly regarding dining and healthcare services. However, areas such as facility maintenance, storage, and opportunities for participation in management require improvement. The findings highlight the critical role of functional status and educational background in shaping satisfaction, suggesting that perceived quality of life is closely linked to personal autonomy and social engagement within the care home environment.

## Introduction

Spain’s population is experiencing rapid demographic aging due to declining fertility rates, increased life expectancy, and advancements in public health (1). Consequently, Spain now has one of the highest aging indices globally (2). This demographic shift necessitates effective psychosocial interventions and policy adjustments to support families, which is likely to increase the demand for residential services.

In 2020, 73.2% of Spanish older adults’ care homes were private compared to 26.8% that were public (3); most of these facilities focus on non-care models that promote independent living (4). In these care homes, between 4 and 6% of adults aged 70 and over reside together (5). Given the specific needs of this cohort, residential care homes should be prioritized as primary institutional responses (6).

According to IMSERSO data from February 2025, 1,494,311 individuals in Spain benefited from state-assisted dependency services, with 419,856 receiving care home or day/night center services through direct provision or financial compensation. Currently, only 181,887 are direct users of public care home services, though demand is projected to grow (7).

Furthermore, the COVID-19 pandemic prompted a critical re-evaluation of care home operations. Residents faced “double confinement”—losing access to both the community and shared internal areas, including rehabilitation programs and family visits. This isolation led to significant cognitive, physical, and emotional deterioration (8–10), highlighting the urgent need to assess quality of life and identify areas for improvement through collaboration among residents, families, staff, and management (11, 12).

Evaluating residential quality requires assessing resident satisfaction and resource allocation. Care homes should function as living spaces centered on relationships, intimacy, social participation, and autonomy rather than mere healthcare centers (13). Consequently, this study explores residents’ satisfaction regarding their living environment, personal relationships, and available services. By analyzing multiple variables, the findings aim to provide meaningful insights for future service provision adjustments.

## Background

### Quality criteria in residential service

Spain’s entry into the European Economic Community required changes to improve social services to optimize product standards and services regardless of whether they were private or public. As part of this transition, AENOR (Spanish Association for Standardization and Certification) was established in 1986. As a member of the International Organization for Standardization (ISO), AENOR coordinates national standards and promotes international ISO standards, such as ISO 9001 (14).

The 2015 version of ISO 9001 introduced an innovative prevention-oriented approach by: (a) performing risk and opportunity analyses to contextualize organizational performance, and (b) adopting a unified management structure to increase stakeholder engagement and client satisfaction.

### Residential services: an environment of quality and friendliness

Within this framework, Standard UNE 158000 was established to promote personal autonomy, specifically through UNE 158101–2015: “Services to promote personal autonomy. Management of care home centers and the integrated day or night care home centers”. This regulatory innovation facilitated a new intervention paradigm where care and quality are inextricably linked. The model prioritizes a Person-Centered Care approach and Preference-Based Model of Care, focusing on individual needs and resident satisfaction as primary objectives (15, 16). Furthermore, architectural design and environment in residential settings for the older adults are very important in the aging process (Person-Environment Fit model by (17)). As Barnes (18) emphasized, it can significantly enhance residents’ well-being and their perception of control. Consequently, variables such as individual preferences, desires, and expectations are central to how residents perceive these resources, and these elements are integrated into the pursuit of excellence in management processes endorsed by these quality standards.

As Martínez Fernández and Barrera (19) noted, quality care models must incorporate and measure interpersonal, psychosocial, and familial dimensions. This approach ensures that evaluation variables align with modern management systems, which integrate professional standards, organizational processes, internal communication, and employee motivation (20).

Since its implementation, the objective of this standard has been to ensure efficient service delivery through the development of quality benchmarks, continuous monitoring, and the evaluation of organizational processes using unified criteria and verification systems. Accordingly, specific indicators (see Table 1) have been included to identify and differentiate existing services while enabling outcome comparisons. To ensure user satisfaction, these indicators focus on three core axes: care provision, the relationship with the environment, and interaction with other services. The significance of these indicators lies in their ability to explicitly define measurable variables for goal achievement, guiding both continuous improvement and comparative analysis. These are key indicators that need to be audited during different phases of the process when evaluating a service.

**Table 1:** UNE indicators to manage care homes.

UNE 158101 indicators to manage care homes (including those with a day/night center)		
Process	Indicator	Periodicity
Start of the service in day and night centers	Percentage of users with a complete initial evaluation during the admittance process	3-monthly
Individual Care Plan (ICP)	Percentage of users with a completed initial ICP	3-monthly
	Percentage of users with a 6-monthly ICP review	6-monthly
Intervention programs	Percentage of users with falls	Monthly
	Percentage of users with uncontrolled departures	
	Percentage of users who participate in socio-cultural activities	
	Percentage of families who participate in activities scheduled for them	
Center’s services	Ratio of incidences during medication	3-monthly
	Percentage of mechanically supported users	
	Percentage of routes with incidences	
Finalizing service	Percentage of leaves for not adapting	3-monthly
Internal process of evaluating service quality	Percentage of satisfied users	Annually
	Percentage of satisfied family relatives	
	Percentage of users and family relatives with complaints	Monthly
Human Resources	Percentage of satisfied workers	Annually
Training	Percentage of workers who had received training	Annually

Mira et al. (21).

## Methods

### Ethical considerations

This study was conducted in accordance with the ethical principles of the Declaration of Helsinki and the good practice guidelines of the University of Zaragoza. Given the non-invasive nature of the research, which consisted of voluntary satisfaction surveys, the study was overseen and approved by S.51 Well-being and Social Capital Research Group (University of Zaragoza). All participants were informed about the study’s objectives and provided their verbal or written informed consent prior to data collection. Participant anonymity and data confidentiality were strictly maintained in compliance with the Spanish Organic Law on Personal Data Protection (LOPD-GDD 3/2018).

### Sample selection

This case study focused on a care home in Sabiñánigo (Aragón, Spain). This private facility is accredited by the Regional Government of Aragón to serve both mobile and dependent users. In 2018, the center had 90 residents, with 50 places designated for severe dependency. This dependent group was excluded from our study, because they lacked adequate cognitive capacity or experienced severe mobility issues that made the interview process difficult for them. Given the study population’s profile, namely the very older adults and associated diseases, our sample included 40 residents.

### Selecting the instrument

Having defined our sample, data collection was the priority, for which direct interviews and questionnaires were completed in the care home. The primary instrument had established reliability, having been used previously by the Social Services of the Alto Gállego Administrative Division. The questionnaire was culturally appropriate, given that it was previously used in this region. After an initial revision and modification phase, it was submitted for evaluation by the multidisciplinary team of the Reference Research Group of the University of Zaragoza and the Regional Government of Aragón, ref. S. 51 Well-being and Social Capital, which relies on an external auditing system (22).

Aligned with UNE 158000 standards (see Table 2), two instruments were developed to collect data, which separately addressed residents or family relatives. Both these questionnaires are made up of three types of questions: dichotomic, scale (numerical and Likert scale) and open options. The collected data also allowed identified socio-demographic aspects of the sample, and these measures were analyzed, along with residents’ degree of satisfaction with:

(a) knowledge or access to information about services (medical, health transport and hospital accompaniment, dining room and laundry) and installations (shared areas and rooms).
(b) ways to participate and relational aspects.
(c) attitudes, degree of agreement, and intensity of feelings.
(d) residents’ tutelage, business management, and communication channels.
(e) personal evaluation of the specific aspects set out in the questionnaire.

**Table 2:** Relation between questionnaire blocks and Standard UNE 158000 quality indicators.

Questionnaire Blocks	UNE 158000 Standards
Block1: Socio-demographic characteristics	Individual Care Plan (ICP)
	Starting the service in day and night centers
Block 2: Installations	Intervention programs
Block 3: Services	Center’s services
	Intervention programs
	Training
Block 4: Participation and relationships in the care home	Internal processes of evaluating service quality

Mira et al. (21).

### Data analysis

To inform the case study, several methods were employed. Descriptive statistics were used to describe the subgroups (frequency data) and response measures related to key variables. Inferential statistics were employed to consider associations between key measures (Pearson correlations) and influences on variables of key interest (regression analysis). Qualitative data were not thematically analyzed but commentaries were used to make sense of the statistical results.

## Results

### Descriptive results

#### Socio-demographic characteristics

Of the 40 people interviewed in the care home, 27 are women (67.5% of cases). The average age of the total sample is 85.5 years, with women averaging approximately 86.35 years and men averaging around 83.73 years. Therefore, 85% of all the participants fall in this age range, which is considered the “fourth age” phase (see Table 3).

**Table 3:** Absolute (N) and relative (%) frequencies by age and gender.

		Total		Men		Women	
		*N*	%	*N*	%	*N*	%
Age (years)	60-69	2	5	1	2.5	1	2.5
	70-79	4	10	2	5	2	5
	80-89	22	55	7	17.5	15	37.5
	90-99	12	30	3	7.5	9	22.5
Total		40	100%	13	32.5%	27	67.5%

The sample analysis reveals that most people are widowed, specifically 55% (with 82% of cases being women). The other residents’ marital status is single with 27.5% and married with 7.5% of cases.

Admittance for 43.2% of cases was in the year prior to this study, while 13.5% had been cared for by this service for more than 5 years.

The lodgings regime data revealed that only 17.5% of all the residents had obtained a state-assisted place, while the rest had been privately admitted. This fact is due mainly to only 22% of our sample being in a ‘dependency’ situation. The interviewed people’s profile was as follows: 42.5% of cases are completely autonomous, and 35.5% sporadically require certain support to perform activities of daily living (ADL) and instrumental ADL.

In summary, the sample consists mainly of women, females aged more than 80 years, widows, or single females with self-care capacities, who came from bordering regions and who were cared for in this care home for more than 1 year.

#### Installations

Our data obtained from residents’ responses indicate that one of the most relevant spaces is their bedroom and areas in close proximity: 80% of residents share a bedroom: 5% with more than one person, and almost 70% do so without their roommate being a family relative. This is particularly important for residents’ degree of satisfaction, who valued the sharing experience as positive in 70% of cases.

The results indicate that although this intimate space is shared, occupants expressed positive perceptions regarding its utility. In addition, the positive view of 90% of those interviewed stands out, especially in reference to furnishings and their maintenance, for which 75% of the sample state “quite good” for the resources related to furnishings in general, and the same for bedroom location, according to 85% of residents.

As opposed to the best valued spaces, such as the TV room and the garden, the space set aside for storing individual belongings (45% of those interviewed state it is not at all or less positive), and the cafeteria are negatively valued. With respect to the cafeteria, 90% of the residents are against the restricted use of vending machines, and 80% value it in the unsatisfactory range.

Regardless of their pros and cons, 97.5% of residents believe that their care home is a very welcoming place.

#### Services

Of all those interviewed, 80% state that the dining room service is good or very good, particularly its mealtimes, and the amount and quality of food served. For 87.5% of those who eat in it, the menu can be consulted the day before, which they perceive as being very satisfactory.

More than 90% of those interviewed gave favorable evaluations for the suitability of their diet and its range, with 65% of cases being satisfied with having special menus available on certain dates.

Less favorably, however, and notably, is that 75% of cases have not participated in, or were allowed to make suggestions, about improvements in care home management.

Another aspect that is relational is that 80% of people were satisfied with the people who sat at their table and the way tables were distributed, and a significant 92% of residents preferred to eat in the dining room rather than in their bedroom.

The best valued service by residents was related to medical health care, with no answers referring to feeling poorly satisfied or not at all satisfied. In addition to this, the results obtained reflect positive figures for accessibility, as indicated by 88% of the beneficiaries, plus optimal opening times for 80% of the sample. It was noted that 52% of those interviewed stated feeling satisfied with the way they were being attended to.

#### Participation and relationships in the care home

Some important variables for seamless participation in the residential environment are the relationship between users and professionals, access to the spaces where these relationships develop, optimal opening times, and clear guidelines regarding their appropriateness and implementation.

Thus, as part of the ordinary protocols of their new co-living environment, this information is given from the time they are admitted to the care home. At the time they were welcomed and admitted, 50% of the residents stated that they were provided with satisfactory information, vs. 25% who believed it was inadequate.

Residents’ communication with professionals in 92.5% of cases is defined as very good. It is also important to know how residents generally perceived their personal relationships with one another, which they point out as being ‘quite’ or very good. What is especially important is the interaction with the person sharing their bedroom, which 87% value as being good or very good.

The findings indicated that participation, and entertainment activities were prioritized according to this order: gymnasium, TV viewing, and walks. Indeed, 80% of residents indicated making, or having made, the most of these sessions, whereas 10% stated not having done so because they had physical issues/diseases that prevented them.

In reference to participants’ satisfaction, 37.5% indicated that entertainment activities were interesting, as opposed to 20% who thought the opposite, and it was stressed that 32.5% held no view on this aspect. For the opening times and durations spent on entertainment or daily tasks, 75% of those interviewed were satisfied with them.

Finally, 65% of the residents stated that they knew the processes and means to participate, while 90% never participated or were unaware of who their representative was during meetings, where complaints and suggestions were made in an open forum.

#### Inferential results

Next, a Pearson’s correlation was employed to examine the associations between variables. This statistical technique was applied to the four domains making up the questionnaire.

Table 4 relates to the services and installations made available in the care home (hairdressing, gymnasium, TV room, garden, maintenance, food, and health care) to one another, which may affect the evaluations that residents made (STATA: pwcorr). The only item that presents positive and statistically significant effects with the care home evaluation is related to the quality of food. The older adults who evaluated the hairdressing service were more satisfied with the TV room and garden, which are places where more socialization takes place. The residents who evaluated the gymnasium highly also stated being very satisfied with the TV room, the garden, and the food and health care that they receive, which are services that help to improve the institutionalized older adults’ health status. Their evaluation of the TV room correlates positively with the evaluations of the garden and the received health care, but inversely with the maintenance of installations. Their evaluation of the care of installations correlates negatively with received health care. From these negative correlations, it is possible to conclude that although residents are happy with personal care and their residential lifestyle, they generally criticize the maintenance of installations more.

**Table 4:** Correlations between satisfaction values

	Evaluation	Hairdressing	Gym	TV room	Garden	Mainten	Food	Health care
Evaluation	1.0000							
Hairdressing	0.3208	1.0000						
Gymnasium	0.1994	0.1684	1.0000					
TV room	0.2148	0.3690*	0.4867*	1.0000				
Garden	0.0832	0.7959*	0.4413*	0.5353*	1.0000			
Maintenance	-0.1661	-0.1188	-0.2275	-0.5239*	-0.0480	1.0000		
Food	0.6276*	0.2663	0.3516*	0.1865	-0.0158	-0.3126	1.0000	
Health care	0.2950	0.0625	0.4716*	0.5617*	0.2449	-0.3763*	0.2641	1.0000

*0.05 (95%) significance level.

### Statistical synthesis of the main results

This last section synthesizes the obtained results. The linear regression method (STATA: Regress) was used in Table 5 to collect the main determinants from the care home evaluations made by its residents. Of the selected variables (gender, age, finished studies, functional status, belonging to the center, and type of bedroom), only statistically significant effects were indicated for finished studies and functional status. The residents who completed Elementary Education (60% of those interviewed) negatively evaluated the care home services compared to those without such studies (30%). As both these groups represent 90% of the interviewed residents, the evidence suggests that the higher their level of finished studies, the lower their care home evaluation. Similarly, functional autonomy correlates negatively with the older adults’ satisfaction with the care home insofar as autonomous residents (43% of those interviewed) negatively evaluated their care home compared to those classified as presenting marked dependency (23%). The few observations and the poor heterogeneity of their responses (e.g., all the residents give a score over 5% for the care home, and 83% give one of 8 or higher on a scale from 1 to 10) advise against using more sophisticated econometric techniques. The linear regression constant generated a value of 6.4, suggesting that residents’ evaluation has a high point of origin regardless of the explanatory variable’s effect.

**Table 5:** Estimating evaluations of the care home

		Description	Beta-Coefficient	*P*- Value	Mean
Gender	Female	Dichotomic variables that inform about residents’ gender	-0.533	0.40	68%
	Male		ref.		33%
Age (years)	Age 60-69	Quantitative variable that informs about residents’ age that takes a value of 1 if between 60 and 69 year, 2 between 70 and 79 years, 3 between 80 and 89 years and 4 between 90 and 99 years	ref.		5%
	Age 70-79		0.155	0.928	10%
	Age 80-89		1.748	0.322	55%
	Age 89-99		2.624	0.165	30%
Finished studies	Read and write	Dichotomic variables that inform about residents’ finished studies. No residents had completed High School/Vocational Training	ref.		30%
	Elemental		-0.999	0.08	60%
	High School or Vocational Training		0.286	0.79	10%
Functional status	Dependent	Dichotomic variables that inform about residents’ functional status	ref.		23%
	Reduced mobility		-0.316	0.64	35%
	Autonomous		-1.381	0.06	43%
Belonging	< 7 months	Dichotomic variables that inform about the time that residents had been at the older adults’s care home	ref.		28%
	7 months - 1 year		0.717	0.44	13%
	1-2 years		-0.584	0.56	10%
	> 2 years		-0.135	0.83	49%
Bedroom	Individual	This variable informs about residents’ type of bedroom	ref.		8%
	Double		0.602	0.54	86%
	Collective		-1.636	0.22	5%
	Constant	Point of origin of residents’ evaluation that the model does not explain	6.398	0.00	

*Evaluation: it indicates the overall evaluation made by the older adults’ care home residents on a scale from 1 to 10 with a mean of 8.5.*

*No evaluation of the care home is below 5.*

## Discussion

### Socio-demographic characteristics

The findings of this study indicated that residents’ functional status influences their degree of perceived satisfaction with the care home’s services and installations. Residents considered to be autonomous felt more satisfied with the residential services.

Although their age and the time since their arrival at the center have poor explanatory importance, they presented unfavorable associations in their evaluations. The survey reflected that older residents are less satisfied with the spaces where vending machines or the gymnasium are located, while those who have spent more time at the care home, think that their belongings are not stored in a safe place, they do not have enough technical material and their responses indicated that they did not like the entertainment activities.

In reference to the socio-demographic characteristics, explanatory importance was identified related to the payment method and if their bedroom was shared or not. Both are associated positively with their bedroom’s habitability conditions. Those residents whose place was private and who share their bedroom feel more satisfied with their personal space.

Feeling well cared for at their care home, participating more in maintenance tasks, being able to choose a table in the dining room, and better evaluating the laundry service, are associated with residents’ particular payment method (non-state-assisted places).

### Installations

#### Shared areas

According to Pino et al. (23), care homes should have different areas (e.g., gardens) that foster communication, and should be equipped with easy access. Joseph et al. (24) stated that these physical environments significantly influence not only residents, but also care home staff by improving quality of life and organizational outcomes.

Nonetheless, it is worth stressing that this case study reports an unfavorable view about access to shared rooms, mainly the room with vending machines or the garden, which affects the evaluation made of the relationship with professionals. Thus, as with previous research [e.g., (25, 26)], some characteristics related to installations (e.g., furnishings, accesses, places for walking) influence and significantly impact perceived quality of life.

Rooms like the TV and Visitors’ rooms are considered clean and comfortable places that allow residents to offer a positive view of the home care installations.

#### Rooms

When evaluating satisfaction with their bedroom, residents highlight factors like their stay, sharing their bedroom or not, and essential resource provisions, e.g., entertainment or laundry services, access to adequate health care, in addition to feeling well cared for.

The data obtained reflected positive evaluation for accessibility to bedrooms and the desirability of having one’s own bathroom. As in the study by Okken et al., (27), the characteristics of the architectural space affect interpersonal communication and intimacy. In addition, other resources were acknowledged, such as provisions for ventilation, lighting, cleanliness, and access to personal objects, own furnishings, and using a personal, preferred bed.

### Services

The key service areas highlighted in this study indicated that the dining room and health resources were considered very important.

As with previous research (26, 28–31), our study evidences that the hours spent by professionals with residents, which are linked with both offering personalized care and performing activities and services, were positively associated with residents’ perceived quality of life. Therefore, the more satisfied they were with these resources, the more likely they were to rate the quality of life positively.

### Dining room service

The residents’ perceptions acknowledged the importance of the food served at the care home. More specifically, they emphasized the need to audit and ensure good dining room service, quality of food, appropriate meal times, and availability of professionals. Residents’ evaluation of the quality of the dining room service was also related to cleaning, the employed space, and the furnishings.

The positive evaluations for knowing the menu beforehand and it being posted on noticeboards were directly related. Furthermore, enjoying different menus on special days was highly valued and directly linked with residents’ degree of satisfaction with the dining room service.

Another aspect, that of being able to choose a table in the dining room, is very positively evaluated, and also reflects an association with the fact that they felt well cared for.

### Healthcare service

Health care is one of the services that is of essential value to residents. The findings indicate that when adequate health care is provided, the degree of life satisfaction is higher, and this is directly linked with the care home being perceived as welcoming.

### Participation and relationships with the care home

To ensure optimal transition into the residential environment and its installations, it is necessary for residents and families to receive correct information upon admittance (32). Clearly articulated information about installations engendered positive evaluation being made of other resources, such as access to rooms and issues related to the menu.

The perceived quality of residents’ stay at the care home, was influenced by the interaction and synergies that take place among those living there and those with residential employees and management personnel. Residents’ satisfaction with the relational area was favorably associated with access to services and rooms, and aligned with residents’ residential views and functional matters like choosing a table in the dining room.

Most people, in this study, were not admitted to the care home through their own initiative, which makes the feeling of belonging and intimacy difficult. According to Pino et al. (22), this situation affects the close links and relationships between residents and their environment. This is clearly present in the findings, because satisfaction with residents’ interaction depends on their previous relationships, e.g., with family relatives. It is also positively associated with a favorable evaluation of access to resources and installations or with their stay at the care home.

Their evaluation of the interaction with their roommate, a very close relationship, favors a positive evaluation of their bedroom and of other participatory behaviors, such as performing maintenance tasks.

Self-evaluations of their care is a key variable to estimate satisfaction with the care home, also for evaluating the quantity of connections that is produces (e.g., 21 variables in this study were significantly correlated). Our findings are in agreement with the study by Acevedo (33), who reported that their perception of residential quality of life is closely linked with personal autonomy, relationships and social participation and intimacy.

## Conclusion

### The inverse correlation: expectations and autonomy

A significant finding of this study is the inverse correlation between residents’ level of education and functional autonomy and their perceived satisfaction. Specifically, residents with higher educational backgrounds and greater autonomy evaluated services less favorably. Although this may seem counter-intuitive, it aligns with the “Person-Centered Care” theory (14). Highly educated residents often possess more critical perspectives and higher expectations regarding management transparency and participation. Our data shows that 90% of residents never participated in management decisions—a gap that likely impacts the more educated cohort most acutely, as they prioritize agency over mere safety.

Regarding functional status, our results support the premise that institutional restrictions more severely affect those who retain their physical and cognitive capabilities. As Barnes (18) emphasized, architectural design and institutional rules are determinants of a resident’s perception of control. In our sample, autonomous residents (42.5%) frequently encountered organizational barriers designed for dependent profiles, such as restricted access to vending machines or the garden. This discrepancy between individual capacity and environmental constraints creates a “Person-Environment Fit” mismatch, generating a perceived loss of control and reducing satisfaction (16).

This disconnection bridges both theoretical frameworks: the institution not only presents physical obstacles but also imposes an ‘environmental press’ that blocks the implementation of Person-Centered Care, preventing care from being based on the preferences and self-determination of the more autonomous residents.

### The relational environment and community integration

Consistent with previous research (25, 29, 34), this study underscores the importance of maintaining a living environment close to the resident’s home and family. This facilitates the psychosocial interactions essential for psychological health and well-being. Consequently, multidimensional evaluations must prioritize residents’ direct perspectives (35–37). Our research suggests that the quality of residential services depends as much on the “relational environment”—including professional dealings and social interactions in shared spaces like dining rooms—as it does on physical equipment (22).

### Institutional diversity and policy implications

Public policies frequently treat the older adults as a homogeneous group, ignoring the diversity inherent in aging. However, our findings demonstrate that education and autonomy act as key predictor variables for satisfaction, necessitating personalized strategies that respect individual differences (33). For instance, the potential use of new technologies, such as virtual reality, offers promising avenues for personalizing care and enhancing cognitive engagement (38).

### Case study specifics: Sabiñánigo

In the Sabiñánigo care home, factors such as payment method and room configuration (shared vs. private) significantly influenced satisfaction. Residents emphasized the importance of personalized, accessible spaces to protect their privacy. The dining service was another highlight; the variety and quality of the food were seen as essential for breaking the monotony of institutional routine. Furthermore, residents valued the “co-living” aspect, reporting positive relationships with roommates and staff (13).

### Areas for improvement: nature and participation

Despite overall positive evaluations, significant room for improvement remains regarding garden access and autonomy to go outside. As the Sabiñánigo facility is located in a rural area with high landscape value, increasing contact with nature could significantly improve emotional health and community integration (39, 40). Finally, while the center has established committees for resident participation, they remain underutilized. To align with international quality standards [ISO; (41)], management must develop more accessible and dynamic participation mechanisms to ensure that residents feel they have a genuine voice in their daily lives.

### Strengths and limitations

#### Strengths

- Standardized Quality Framework: The study’s evaluation criteria are strictly aligned with international and national quality standards, including ISO 9001 and UNE 158101–2015.
- Person-Centered Focus: The research prioritizes the Person-Centered Care approach, emphasizing individual autonomy, social participation, and the quality of the relational environment rather than purely clinical metrics.
- Methodological Rigor: Data collection utilized an instrument with established regional reliability, further validated by a multidisciplinary team from the University of Zaragoza and an external auditing system.
- Contextual Depth: As a case study in Sabiñánigo, the research provides a detailed analysis of how specific environmental factors, such as rural landscape value and dining service quality, influence the emotional well-being of residents.

#### Limitations

- Sample Constraints: The study is limited by a small sample size of N = 40 participants, which restricted the use of more complex statistical techniques.
- Selection Bias: Residents with severe dependency or cognitive impairments were excluded from the sample due to communication difficulties, meaning the results do not capture the perspectives of the most vulnerable residents.
- Generalizability: Because this research focuses on a single private facility in a specific rural region, the findings may not be fully representative of public care homes or urban residential centers.
- Response Heterogeneity: A potential ceiling effect was observed, as 83% of residents reported satisfaction scores of 8 or higher, indicating a lack of diversity in the responses and possible social desirability bias.

## Data Availability

The raw data supporting the conclusions of this article will be made available by the authors, without undue reservation.

## References

[1] González Río MJ San Miguel del Hoy B. El envejecimiento de la población española y sus consecuencias sociales. Alternativas: Cuadernos de trabajo social. (2001) 9:19–45. doi: 10.14198/ALTERN2001.9.2

[2] Domínguez A López C Picardo JM. Envejecer en una residencia de ancianos en España: una revisión integradora. Gerokomos. (2023) 34:176–82. Available at: http://scielo.isciii.es/scielo.php?script=sci_arttext&pid=S1134-928X2023000300005&lng=es&nrm=iso

[3] Abellán A Aceituno MP Ramiro D Castillo AB. Estadísticas sobre residencias: distribución de centros y plazas residenciales por provincia. Informes Envejecimiento en Red. (2021) 27:24. Available at: http://envejecimiento.csic.es/documentos/enred-estadisticasresidencias2020.pdf

[4] Picardo JM. COVID-19 en residencias de mayores: una asignatura pendiente. Enferm Clin. (2020) 31:S117–9. doi: 10.1016/j.enfcli.2020.05.015, 34629858 PMC7241382

[5] Tarazona FJ Martínez N Vidán MT García JA. COVID-19, adulto mayor y edadismo. Rev Esp Geriatr Gerontol. (2020) 55:191–2. doi: 10.1016/j.regg.2020.04.001, 32386947 PMC7188650

[6] Esteban L Rodríguez JA. Situaciones de dependencia en personas mayores en las residencias de ancianos en España. Ene. (2015) 9. doi: 10.4321/S1988-348X2015000200007

[7] Instituto de Mayores y Servicios Sociales (2025). Información estadística destacada del SAAD. Available online at: https://imserso.es/el-imserso/documentacion/estadisticas/sistema-autonomia-atencion-dependencia-saad/estadisticas-mensual (Accessed February 3, 2025).

[8] Pinazo S. Impacto psicosocial de la COVID-19 en las personas mayores. Rev Esp Geriatr Gerontol. (2020) 55:249–52. doi: 10.1016/j.regg.2020.05.006, 32741601 PMC7266768

[9] Plagg B Engl A Piccoliori G Eisendle K. Prolonged social isolation of the older adults during COVID-19: between benefit and damage. Arch Gerontol Geriatr. (2020) 89:104086. doi: 10.1016/j.archger.2020.104086, 32388336 PMC7196375

[10] Sepúlveda W Rodríguez I Pérez P Ganz F Torralba R Oliveira DV . Impact of social isolation due to COVID-19 on health in older people. J Nutrition Health Aging. (2020) 24:938–47. doi: 10.1007/s12603-020-1469-2 33155618 PMC7597423

[11] Authors. What do families value most about the care home where their older adult relatives live? Front Public Health. (2024) 12:1338649. doi: 10.3389/fpubh.2024.1338649, 39175896 PMC11340505

[12] Avidor S Ayalon L. I didn't meet my mother; I saw my mother: the challenges facing long-term care residents and their families in the age of COVID-19. J Appl Gerontol. (2022) 41:22–9. doi: 10.1177/07334648211037099, 34365855

[13] Kabo FW. A model of potential encounters in the workplace: the relationships of Homophily, spatial distance, organizational structure, and perceived networks. Environ Behav. (2017) 49:638–62. doi: 10.1177/0013916516658501

[14] Asociación Española de Normalización y Certificación AENOR. Norma UNE 158101 de Servicios para la promoción de la autonomía personal, gestión de centros residenciales y centros residenciales con centro de día o centro de noche integrado. Madrid: AENOR (2007).

[15] Martínez Rodríguez T. (2015). La atención centrada en la persona en los servicios gerontológicos. Universidad de Oviedo. Available online at: http://digibuo.uniovi.es/dspace/bitstream/10651/33762/1/TD_TeresaMartinez.pdf (Accessed January 28, 2026).

[16] Silveira MIB Hembecker PK Scharan KO Moser ADL. Environmental barriers and facilitators: impacts on the functionality of institutionalized and community-dwelling older adults. Revista Brasileira de Geriatria e Gerontologia. (2025) 28:e240181. doi: 10.1590/1981-22562025028.240181.en

[17] Lawton MP Nahemow L. Ecology and the aging process In: Eisdorfer C Lawton MP, editors. The psychology of adult development and aging. Washington: American Psychological Association (1973). 619–74.

[18] Barnes S. Space, choice and control, and quality of life in care settings for older people. Environ Behav. (2006) 38:589–604. doi: 10.1177/0013916505281578

[19] Martínez Fernández R Barrera E. Fortalezas y debilidades de los sistemas de gestión de la calidad implantados en los centros de personas mayores en España. Cultura de los Cuidados. (2021) 25:268–285. doi: 10.14198/cuid.2021.61.17

[20] Saut AM Tobal Berssaneti F Moreno MC. Evaluating the impact of accreditation on Brazilian healthcare organizations. Int J Qual Health Care. (2017) 29:713–21. doi: 10.1093/intqhc/mzx094, 28992152

[21] Mira DC Martínez J. Valoración de la satisfacción de los servicios prestados en la residencia Alto Gállego. Zaragoza: BYCS (2019).

[22] Authors (2023). El bienestar individual, familiar y colectivo: acceso y uso de recursos socio-sanitarios. [Doctoral thesis, Universidad de Zaragoza].

[23] Pino MR Crespo JM Portela J. Descripción de los elementos espaciales en residencias de ancianos. Revista de Investigación en Educación. (2010) 7:61–71. Available at: https://revistas.uvigo.es/index.php/reined/article/view/1851

[24] Joseph A Choi YS Quan XB. Impact of the physical environment of residential health, care, and support facilities (RHCSF) on staff and residents: a systematic review of the literature. Environ Behav. (2016) 48:1203–41. doi: 10.1177/0013916515597027

[25] Leung M Yu J Chong MLA. Impact of facilities management on the quality of life for the older adults in care and attention homes. Indoor Built Environ. (2017) 26:1070–90. doi: 10.1177/1420326X16662697

[26] Rodríguez A Martín M d C de la Fuente YM Jiménez JJ. Influence of facility characteristics on the quality of life of older adult residents. SAGE Open. (2023) 13. doi: 10.1177/21582440231201001

[27] Okken V van Rompay T Pruyn A. Room to move: on spatial constraints and self-disclosure during intimate conversations. Environ Behav. (2013) 45:737–60. doi: 10.1177/0013916512444780

[28] Abrahamson K Lewis T Perkins A Clark D Nazir A Arling G. The influence of cognitive impairment, special care unit placement, and nursing facility characteristics on resident quality of life. J Aging Health. (2013) 25:574–88. doi: 10.1177/0898264313480240, 23511654 PMC4059007

[29] Kehyayan V Hirdes JP Tyas SL Stolee P. Predictors of long-term care facility residents’ self-reported quality of life with individual and facility characteristics in Canada. J Aging Health. (2016) 28:503–29. doi: 10.1177/0898264315594138, 26148943

[30] Shippee TP Henning-Smith C Kane RL Lewis T. Resident- and facility-level predictors of quality of life in long-term care. The Gerontologist. (2015) 55:643–55. doi: 10.1093/geront/gnt148, 24352532 PMC4542585

[31] Shippee TP Hong H Henning-Smith C Kane RL. Longitudinal changes in nursing home resident-reported quality of life. Res Aging. (2015) 37:555–80. doi: 10.1177/0164027514545975, 25651583 PMC9907636

[32] Ball MM Perkins MM Whittington FJ Connell BR Hollingsworth C King SV . Managing decline in assisted living. J Gerontol Series B. (2004) 59:202–12. doi: 10.1093/geronb/59.4.s202 15294924

[33] Acevedo E. Quality of life of institutionalized older adults people in nursing homes: a quantitative analysis. European J Investigation Health, Psychol Educ. (2014) 4:225–34. doi: 10.1989/ejihpe.v4i3.70

[34] Leung M Famakin IO Olomolaiye P. Effect of facilities management components on the quality of life of Chinese older adults in care and attention homes. Facilities. (2017) 35:270–85. doi: 10.1108/F-03-2016-0032

[35] Degenholtz HB Kane RA Kane RL Bershadsky B Kling KC. Predicting nursing facility residents’ quality of life using external indicators. Health Serv Res. (2006) 41:335–56. doi: 10.1111/j.1475-6773.2005.00494.x, 16584452 PMC1702527

[36] Duan Y Mueller CA Yu F Talley KM Shippee TP. The relationships of nursing home culture change practices with resident quality of life and family satisfaction. Res Aging. (2021) 44:174–85. doi: 10.1177/01640275211012652, 33973498 PMC9126004

[37] Finnema E De Lange J Droe Es RM Ribbe M Van Tilbuerg W. The quality of nursing home care: do the opinions of family members change after implementation of emotion-oriented care? J Adv Nurs. (2001) 35:728–40. doi: 10.1046/j.1365-2648.2001.01905.x, 11529975

[38] Tanja-Dijkstra K Pahl S White MP Auvray M Stone RJ Andrade J . The Soothing Sea: a virtual coastal walk can reduce experienced and recollected pain. Environ Behav. (2018) 50:599–625. doi: 10.1177/0013916517710077, 29899576 PMC5992839

[39] French S Wood L Foster SA Giles B Frank L Learnihan V. Sense of community and its association with the neighborhood built environment. Environ Behav. (2014) 46:677–97. doi: 10.1177/0013916512469098

[40] Van Heezik Y Freeman C Buttery Y Waters DL. Factors affecting the extent and quality of nature engagement of older adults living in a range of home types. Environ Behav. (2020) 52:799–829. doi: 10.1177/0013916518821148

[41] Authors. Satisfacción del usuario del Servicio de Ayuda a Domicilio para personas dependientes: Estudio de un vecindario urbano. Acciones e Investigaciones Sociales. (2020) 41:137–70. doi: 10.26754/ojs_ais/ais.2020415123

[42] Asociación Española de Normalización y Certificación AENOR. Norma UNE 158101 de Servicios para la promoción de la autonomía personal, gestión de centros residenciales y centros residenciales con centro de día o centro de noche integrado. Madrid: AENOR (2007).

[43] Mira DC Martínez J Author. Valoración de la satisfacción de los servicios prestados en la residencia Alto Gállego. Zaragoza: BYCS (2019).

